# The Risk of Chronic Pancreatitis in Patients with Psoriasis: A Population-Based Cohort Study

**DOI:** 10.1371/journal.pone.0160041

**Published:** 2016-07-28

**Authors:** Hsien-Yi Chiu, Chi-Feng Hsieh, Yi-Ting Chiang, Weng-Foung Huang, Tsen-Fang Tsai

**Affiliations:** 1 Institute of Biomedical Engineering, College of Medicine and College of Engineering, National Taiwan University, Taipei, Taiwan; 2 Department of Dermatology, National Taiwan University Hospital Hsin-Chu Branch, Hsinchu, Taiwan; 3 Department of Dermatology, National Taiwan University Hospital and National Taiwan University College of Medicine, Taipei, Taiwan; 4 Institute of Health and Welfare Policy, National Yang-Ming University, Taipei, Taiwan; University of Szeged, HUNGARY

## Abstract

**Background:**

Psoriasis is a chronic systemic inflammatory disorder, and studies have revealed its association with a variety of comorbidities. However, the risk of chronic pancreatitis (CP) in psoriasis has not been studied. This study aimed to investigate the risk of CP among patients with psoriasis.

**Methods:**

Using the Taiwan National Health Insurance Research Database, this population-based cohort study enrolled 48430 patients with psoriasis and 193720 subjects without psoriasis. Stratified Cox proportional hazards models were used to compare the risks of CP between the patients with and without psoriasis.

**Results:**

The incidence of CP was 0.61 per 1000 person-years in patients with psoriasis and 0.34 per 1000 person-years in controls during a mean 6.6-year follow-up period. Before adjustment, patients with psoriasis had a significantly higher risk of CP (crude hazard ratio (HR) = 1.81; 95% confidence interval (CI) = 1.53–2.15), and the risk remained significantly higher after adjustments for gender, age group, medications, and comorbidities (adjusted HR (aHR) = 1.76; 95% CI = 1.47–2.10). All psoriasis patient subgroups other than those with arthritis, including those with mild and severe psoriasis and those without arthritis, had significantly increased aHRs for CP, and the risk increased with increasing psoriasis severity. Psoriasis patients taking nonsteroidal anti-inflammatory drugs (aHR = 0.33; 95% CI = 0.22–0.49) and methotrexate (aHR = 0.28; 95% CI = 0.12–0.64) had a lower risk of developing CP after adjustments.

**Conclusions:**

Psoriasis is associated with a significantly increased risk of CP. The results of our study call for more research to provide additional insight into the relationship between psoriasis and CP.

## Introduction

Psoriasis, a chronic inflammatory T-cell-mediated disease, affects 2–3% of the general population and was considered a disease limited to the skin and joints.[1–4] Recent research has emphasized that psoriasis is a multisystemic disease associated with a variety of comorbidities, such as cardiovascular disease, diabetes, and chronic renal diseases.[5–10] However, only few small studies or case reports have been published regarding the pancreas dysfunction or acute pancreatitis in psoriasis.[11–13] And the study investigating the risk of chronic pancreatitis (CP) in psoriasis is lacking.

CP, an irreversible inflammatory disease of the pancreas, is characterized by chronic inflammatory cell infiltration and acinar cell degeneration, leading to progressive destruction of exocrine and endocrine tissue and fibrosis.[14] Moreover, patients with CP have a higher mortality and morbidity than the general population.[14–16]

Chronic pancreatitis may have an insidious onset or develop following episodes of acute pancreatitis. Emerging work had strongly suggested autoimmune diseases, including rheumatoid arthritis, Sjogren’s syndrome, and inflammatory bowel disease, are frequently associated with autoimmune pancreatitis.[17–21] Acute pancreatitis has also been related to long-term use of immunosuppressives.[22–25] Moreover, there is a shared common factor between CP and psoriasis, including hyperlipidemia, chronic renal failure, and cigarette smoke.[8, 14] Therefore, we assessed the risk of CP in a large nationally representative, population-based cohort of Chinese patients with psoriasis from Taiwan.

## Materials and Methods

### Study Design

The design of this study was a retrospectively cohort study. We took advantage of population-based claim data from National Health Insurance (NHI) to identify psoriasis patients during 2004–2006. We matched these patients with general non-psoriasis population during 2004–2006 by age and gender, and follow up their incidence of CP for at least five years. Cohort entry of patients with psoriasis was the date when psoriasis was first diagnosed; matched subjects without psoriasis were assigned the same entry date. This protocol was approved by the Investigational Research Bureau of National Taiwan University Hospital Hsin-Chu Branch (103-024-E).

### Data sources

The Taiwan National Health Insurance Research Database (NHIRD) is an administrative database, compiled by the Taiwan National Health Research Institutes, which is widely used in academic studies. Because the Taiwan NHI program covers nearly 100% of approximately 23 million Taiwan residents, the NHIRD stores data from a very large number of individuals. This compulsory health insurance program provides comprehensive benefits, including ambulatory care and inpatient services. Therefore, NHIRD holds detailed information that includes registration files, demographic data, clinical visits and hospitalizations, diagnostic codes, prescription profiles, and procedures and surgeries. Patients’ identities are encrypted to protect privacy. In order to ensure the accuracy and reliability of coding, the Bureau of the NHI of Taiwan performs random crosschecking and peer reviews, requires justification for any claim by another independent physician, and imposes heavy fines for false claims, overcharging, or malpractice for fraudulent claims. Thus, the NHIRD data are generally accepted as accurate and reliable.

This study used two sources in the NHIRD to identify patients with psoriasis and matched subjects: 1) a dataset that included claims from all patients with psoriasis in Taiwan from 2003 to 2011, and 2) the Longitudinal Health Insurance Database (LHID) 2000, a longitudinal database containing all healthcare information on a representative community-based Taiwan population from 2000 to 2011.

### Study population

The cohort of patients with psoriasis included all individuals diagnosed with psoriasis (International Classification of Disease, Ninth Revision, Clinical Modification [ICD-9-CM] codes 696.0, 696.1, 696.8) between January 1, 2004, and December 31, 2006. For data accuracy, only individuals diagnosed twice with psoriasis by dermatologists during ambulatory visits or inpatient care were included. The date of the first psoriasis diagnosis was designated as the index date from which follow-up began. We further excluded subjects younger than 18 years, patients who had a history of chronic or acute pancreatitis (ICD-9-CM codes 577.0 and 577.1), or pancreatic cancer (ICD-9 code 157) before the index date.

**Fig 1:**
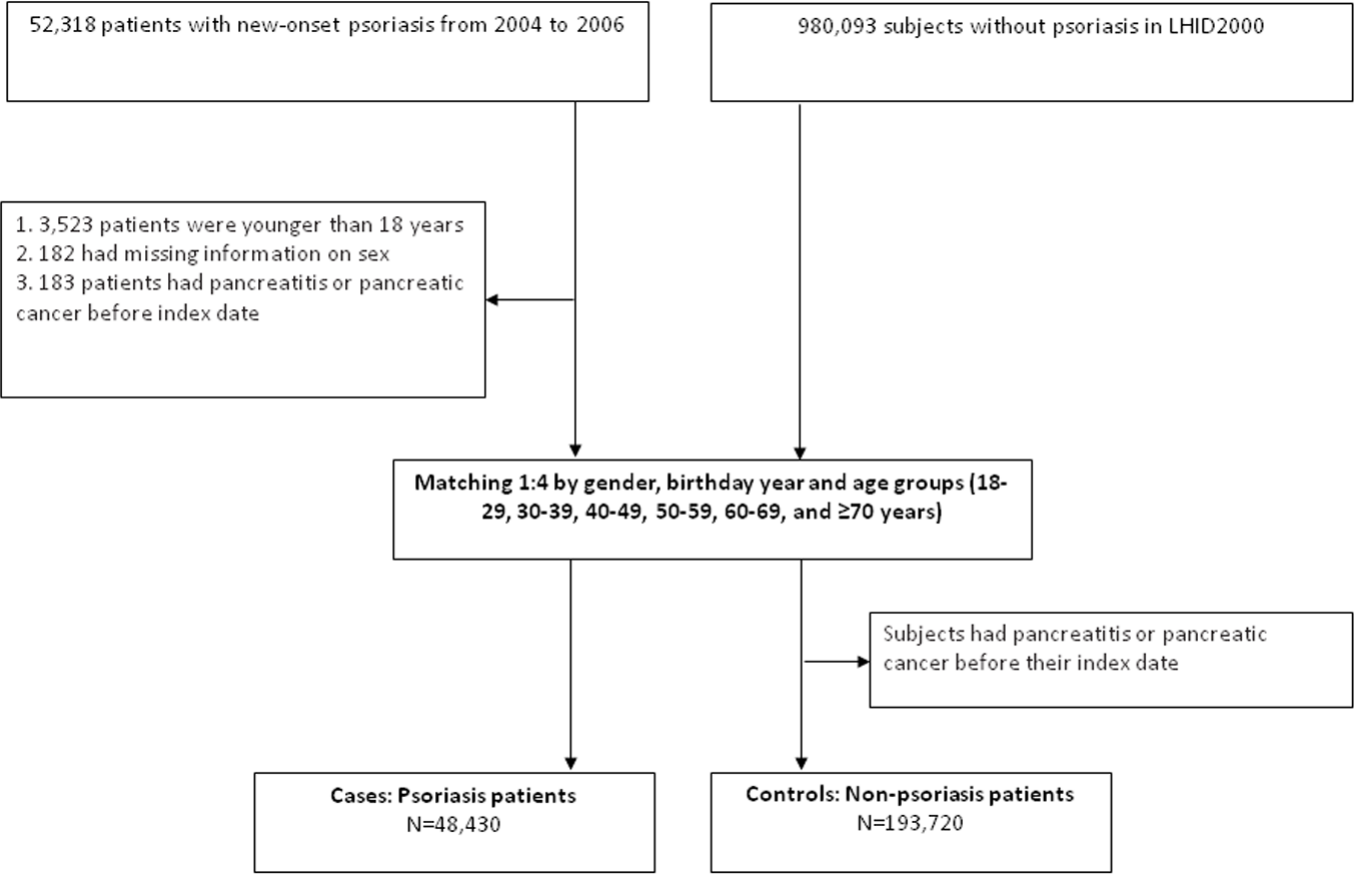
Flowchart. Selection of the study population.

We extracted the control subjects from the LHID2000. We first excluded every subject who had received a diagnosis of psoriasis in the LHID2000. For each psoriasis patient, four subjects without psoriasis, matched on age and gender, were randomly selected from the LHID2000. The index date for the control cohort was corresponding to that given by each matched case. We also ensured that subjects selected for the control cohort had never been given a diagnosis of pancreatitis or pancreatic cancer before their index date (**Fig 1**).

Psoriasis patients were classified into groups with severe and mild psoriasis. Criteria defining severe psoriasis were having received systemic antipsoriatic therapy and/or phototherapy at least once during the first 3 years of follow-up. Patients who had never received systemic antipsoriatic therapy and/or phototherapy were designated as having mild psoriasis. Patients were also classified into groups with psoriatic arthritis (PsA), which was defined as those with at least 2 claims using ICD-9-CM code 696.0 during the same period; otherwise, patients were considered to have psoriasis without PsA.

### Outcomes

The primary outcome was defined as first ambulatory visit or hospitalizations for CP (ICD-9-CM code 577.1), no matter the patients were alive or passed away after the CP event. All subjects were followed up from the index date until the incidence of CP or censored at the end of study period or the date of disenrollment (which is due to death most of the time).

### Statistical analysis

The main analysis assessed time to the first CP. Patients who did not have CP before death or the end of the study were censored. We used the chi-square test to examine associations between the two CP exposure groups (Y/N) for categorical variables. Stratified Cox proportional hazards models were used to estimate the risk of CP. The multivariate models included adjustments for the following: (1) model 1: demographic variables (age groups, gender), cardiovascular conditions, and medical history (cholelithiasis (ICD-9-CM 574), hypertension (ICD-9-CM 362.11, 401–405, and 437. 2), hypertriglyceridemia (ICD-9-CM 272.1), diabetes (ICD-9-CM 250.xx), hepatitis B (ICD-9-CM 070.2, 070.3, and V02.61) and C (ICD-9-CM 070.41, 070.44, 070.51, 070.54, V02.62, and 070.7), cardiovascular disease (ICD-9-CM 410–429), obesity (ICD-9-CM 278.0x), alcohol-related illness (ICD-9-CM 291, 303, 305.0, 571.0, 571.1, 571.2, 571.3, 790.3, and V11.3), chronic obstructive pulmonary disease (ICD-9-CM 491, 492, and 496), and tobacco use disorder (ICD-9-CM 305.1)). (2) model 2: those variables mentioned above plus medications used (methotrexate, cyclosporine, azathioprine, hydroxyurea, cyclophosphamide, acitretin, NSAID, etanercept, and adalimumab). Moreover, because we did not want to lose observations of treated subjects and wanted an interpretable overall treatment effect, we further used propensity score weighting to re-analyze the results. Weighting would allow us to estimate both the average treatment effect of the entire population (ATE weighting) and that of the treated (ATT weighting).[26] The ATE is “the average effect of moving an entire population from untreated to treated.” For the ATE weighing, we applied stabilized ATE weighting because it can deal with the extreme values of propensity scores. The ATT is “the average effect of treatment on those subjects who ultimately received the treatment.”[26]

In subgroup analyses, each subgroup compared with their matched subjects, and risks analyzed as above. Multiple sensitivity analyses were performed to test the underlying assumptions of our primary analysis and the robustness of our findings. All *p*-values were two sided, with *p*<0.05 considered statistically significant. All statistical analyses were performed using SAS version 9.2.

## Results

**Table 1** shows the sociodemographic characteristics in subjects with and without psoriasis. Patients with psoriasis were more likely to have diabetes (10.92% vs. 7.48%, P < 0.001), hypertension (22.0% vs. 16.3%, P < 0. 001), hypertriglyceridemia (0.99% vs. 0.68%, P < 0.001), cardiovascular disease (11.65% vs. 8.63%, P < 0.001), alcohol-related illness (1.55% vs. 0.92%, P < 0.001), obesity (0.37% vs. 0.20%, P < 0.001), chronic obstructive pulmonary disease (6.72% vs. 4.62%, P < 0.001), hepatitis C (0.94% vs. 0.57%, P < 0.001), and cholelithiasis (1.26% vs. 0.99%, P < 0.001) than people without psoriasis at the baseline (**Table 1**).

**Table 1:** Distribution of gender, age, comorbidity, medications in individuals with and without psoriasis.

Characteristic	No.(%) of individiuals		
	Subjects with psoriasis n = 48,430	Subjects without psoriasis n = 193,720	p-value
**Sex**			
Female	20,069 (41.44%)	80,276 (41.44%)	1.00
Male	28,361 (58.56%)	113,444 (58.56%)	
**Age Group**			
18–29	10,367 (21.41%)	41,468 (21.41%)	1.00
30–39	8,222 (16.98%)	32,888 (16.98%)	
40–49	9,283 (19.17%)	37,132 (19.17%)	
50–59	7,966 (16.45%)	31,864 (16.45%)	
60–69	5,481 (11.32%)	21,924 (11.32%)	
≧70	7,111 (14.68%)	28,444 (14.68%)	
**Comorbidity**			
Hypertension	10,655 (22.00%)	31,517 (16.27%)	<0.0001
Diabetes	5,287 (10.92%)	14,491 (7.48%)	<0.0001
Hypertriglyceridemia	481 (0.99%)	1,323 (0.68%)	<0.0001
Obesity	178 (0.37%)	389 (0.20%)	<0.0001
Cardiovascular disease	5,644 (11.65%)	16,716 (8.63%)	<0.0001
Alcoholrelated illness	752 (1.55%)	1,788 (0.92%)	<0.0001
Cholelithiasis	609 (1.26%)	1,918 (0.99%)	<0.0001
Chronic obstructive pulmonary disease	3,256 (6.72%)	8,946 (4.62%)	<0.0001
Hepatitis B	101 (0.21%)	361 (0.19%)	0.32
Hepatitis C	456 (0.94%)	1,102 (0.57%)	<0.0001
Tobacco use disorder	259 (0.53%)	674 (0.35%)	<0.0001
Chronic kidney disease	2,164 (4.47%)	6,214 (3.21%)	<0.0001
**Drug**			
Methotrexate	4,950 (10.22%)	865 (0.45%)	<0.0001
Cyclosporine	711 (1.47%)	191 (0.10%)	<0.0001
Azathioprine	370 (0.76%)	255 (0.13%)	<0.0001
Hydroxyurea	69 (0.14%)	105 (0.05%)	<0.0001
Cyclophosphamide	179 (0.37%)	465 (0.24%)	<0.0001
Acitretin	2,474 (5.11%)	82 (0.04%)	<0.0001
NSAID	46,040 (95.07%)	165,263 (85.31%)	<0.0001
Etanercept	137 (0.28%)	57 (0.03%)	<0.0001
Adalimumab	97 (0.2%)	32 (0.02%)	<0.0001

The incidence of CP during a mean 6.6-year follow-up period was 0.61 per 1000 person-years in patients with psoriasis and 0.34 per 1000 person-years in controls. Before adjustment, patients with psoriasis had a significantly higher risk of CP (crude HR 1.81; 95% CI 1.53–2.15), which remained significant after adjustment (adjusted HR [aHR] 1.76; 95% CI 1.47–2.10) (**Table 2**). We further grouped patients with psoriasis into those with mild or severe psoriasis and those with or without PsA. All psoriasis patient subgroups, including those with mild (aHR 1.81; 95% CI 1.50–2.19) and severe (aHR 1.68; 95% CI 1.02–2.78) psoriasis and those without arthritis (aHR 1.94; 95% CI 1.60–2.37), had significantly increased aHRs for CP, except those with arthritis (aHR 1.20; 95% CI 0.81–1.79). (**Table 2**).

**Table 2:** Incidence of and hazard ratios (HRs) for chronic pancreatitis in subjects with psoriasis (patients) and without psoriasis (controls).

	All psoriasis		Arthritis		Non-Arthritis		Mild Psoriasis		Severe psoriasis	
Variable	Control (n = 193,720)	Patients (n = 48,430)	Control (n = 60,316)	Patients (n = 15,079)	Control (n = 133,404)	Patients (n = 33,351)	Control (n = 151,972)	Patients (n = 37,993)	Control (n = 41,748)	Patients (n = 10,437)
**Follow-up time (years)**										
Mean(SD)	6.59(0.89)	6.58 (0.91)	6.58 (0.88)	6.58 (0.89)	6.59 (0.89)	6.58 (0.92)	6.56 (0.89)	6.55 (0.91)	6.68(0.89)	6.68 (0.90)
Median(Q1,Q3)	6.66 (1.54)	6.66 (1.54)	6.62 (1.53)	6.62 (1.53)	6.67 (1.54)	6.67 (1.55)	6.62 (1.53)	6.62 (1.53)	6.78(1.55)	6.78 (1.55)
No of person years	1,276,332	318,729	396,753	99,155	879,580	219,573	997,367	249,013	278,965	69,716
No(%) of new cases of chronic pancreatitis	433(0.22%)	196 (0.40%)	119 (0.20%)	36(0.24%)	314 (0.24%)	160(0.48%)	338 (0.22%)	164(0.43%)	95(0.23%)	32(0.31%)
Incidence per 1000 person years(95% CI)	0.34(0.31–0.37)	0.61(0.53–0.71)	0.30(0.25–0.36)	0.36 (0.26–0.50)	0.36 (0.32–0.40)	0.73(0.62–0.85)	0.34 (0.30–0.38)	0.66(0.57–0.77)	0.34(0.28–0.42)	0.46(0.32–0.65)
**Hazard ratio (95% CI) for incident CP**										
Unadjusted	1(ref)	1.81(1.53–2.15)^‡^	1(ref)	1.21(0.83–1.76)	1(ref)	2.04(1.69–2.47)^‡^	1(ref)	1.94(1.61–2.34)^‡^	1(ref)	1.35(0.90–2.01)
Model 1^a^	1(ref)	1.64(1.39–1.95)^‡^	1(ref)	1.04(0.72–1.52)	1(ref)	1.87(1.55–2.27)^‡^	1(ref)	1.77(1.46–2.13)^‡^	1(ref)	1.22(0.81–1.82)
Adjusted HR										
Model 2^b^	1(ref)	1.76(1.47–2.10)^‡^	1(ref)	1.20(0.81–1.79)	1(ref)	1.94(1.60–2.37)^‡^	1(ref)	1.81(1.50–2.19)^‡^	1(ref)	1.68(1.02–2.78)^†^
Adjusted HR										

Abbreviations: CI, confidence interval; CP, chronic pancreatitis; HR, hazard ratio; IQR, interquartile range; SD, standard deviation.

†p < 0.05

^‡^p < 0.001 for comparison between patients with psoriasis and non-psoriasis.

^a^Model 1 is adjusted for gender, age group, and all comorbidities listed.

^b^Model 2 is adjusted for gender, age group, medications, and all comorbidities listed.

Next, we investigated the association between concomitant drug and the risk of developing CP (**Table 3**). Our data analysis compared psoriasis patients who had used and those who had not used a specific anti-psoriatic drug. Exposure to non-steroidal anti-inflammatory drugs (NSAIDs) (aHR 0.33; 95% CI 0.22–0.49) and methotrexate (aHR 0.28; 95% CI 0.12–0.64) therapy were associated with a decreased risk of CP after adjustment for age, gender, comorbidities, and other medications. Cyclopsorine, azathioprine, cyclophosphamide, acitretin, and hydroxyurea use didn’t show a significant association with the risk of CP.

**Table 3:** Risks of chronic pancreatitis among psoriasis patients treated with medications.

Medication	Crude HR(95%CI)	Adjusted HR^a^(95%CI)
Methotrexate	0.27 (0.12–0.62)^†^	0.28 (0.12–0.64)^†^
Cyclosporine	0.68 (0.17–2.74)	1.31 (0.30–5.73)
Azathioprine	1.33 (0.33–5.36)	2.20 (0.53–9.21)
Hydroxyurea	3.59 (0.50–25.64)	4.32 (0.60–31.12)
Cyclophosphamide	1.35 (0.19–9.65)	1.76 (0.23–13.21)
Acitretin	0.77 (0.38–1.58)	0.88 (0.43–1.82)
NSAID	0.30 (0.20–0.45)^‡^	0.33 (0.22–0.49)^‡^

Abbreviations: CI, confidence interval; HR, hazard ratio; NSAIDs, nonsteroidal anti-inflammatory drugs.

†p < 0.05

^‡^p < 0.001 for comparison between patients with psoriasis and non-psoriasis.

^a^Adjusted for gender, age, other medications, and all comorbidities listed.

To further verify the robustness of the primary results, we performed sensitivity analysis (**Table 4**). Primary model with propensity score weighting was used to control for selection bias between psoriasis and comparison groups. The results were similar to the primary analyses. Moreover, patients with severe psoriasis had a higher aHR for CP than those with mild psoriasis (aHR 2.37 vs. 2.06) (**Table A in S1 File**). To estimate the impact of alcohol consumption on the risk of CP in patients with psoriasis, we excluded patients with alcohol related illness prior to study start, which did not attenuate the observed association between psoriasis and risk of CP. We also used a more specific outcome definition of CP by combining the ICD-9-CM diagnostic code with imaging studies, such as abdominal ultrasonography, computed tomography, or magnetic resonance imaging. Similar results were also apparent for the risk of CP in psoriasis.

**Table 4:** Sensitivity analyses of the risk of chronic pancreatitis in psoriasis compared with the reference control cohort.

	Crude HR (95% CI)	aHR (95% CI)^a^
Primary model	1.81 (1.53–2.15)^‡^	1.76 (1.47–2.10)^‡^
Primary model with exclusion of alcohol related illness	1.72 (1.44–2.06)^‡^	1.73 (1.44–2.09)^‡^
Propensity score weighting model (ATE)	1.67 (1.41–1.98)^‡^	1.89 (1.61–2.22)^‡^
Propensity score weighting model (ATE) with exclusion of alcohol related illness	2.03 (1.72–2.41)^‡^	1.91 (1.61–2.25)^‡^
Primary model with modified definition of the outcome^b^	2.08 (1.73–2.50)^‡^	2.03 (1.67–2.45)^‡^

Abbreviations: aHR, adjusted hazard ratio; ATE: average treatment effect; HR: hazard ratio; CI: confidence interval

^‡^p < 0.001 for comparison between patients with psoriasis and non-psoriasis.

^a^Adjusted for gender, age group, medications, and all comorbidities listed.

^b^ Refers to having ICD-9-CM code of chronic pancreatitis plus at least one of the following: undergoing amylase/lipase exams within 6 months prior to the diagnosis of chronic pancreatitis or receiving medical imaging studies such as abdominal ultrasonography, computed tomography, or magnetic resonance imaging within 6 months before and after the diagnosis of chronic pancreatitis.

## Discussion

CP is meant to be an irreversible process of damage to the pancreatic tissue and thus eventually causes a variety of morbidity, including debilitating pain, progression to diabetes, and pancreatic cancer.[16] Moreover, patients with CP had a 4-fold higher mortality than the general population.[15] A recent observational study using the UK General Practice Research Database showed that psoriasis patients have an increased risk of pancreatic cancer (Incidence rate ratios 2.20; 95%CI 1.18–4.09) and the risk increased with duration of psoriasis.[27] Although the actual mechanisms contributing to the association between CP and psoriasis remains to be elucidated, the role of the immune system and inflammatory process might be the pathogenic link between the two conditions. In both mouse and human studies, inflammation has been shown to be a precursor to CP.[28–29] The pivotal mediators of inflammation in psoriasis, including tumor necrosis factor (TNF) -α, interleukin (IL)-1, IL-17, and IL-18, are also overexpressed in patients with pancreatitis and their expressions are related to the severity of pancreatic destruction and eventual mortality.[28, 30–33] Mews et al. showed that persistent activation of the stellate pancreatic cells by TNF-α and IL-1 could be a factor in the progression of CP.[34–35] In murine models, TNF-α has been shown to induce severe pancreatitis and be involved in subsequent pancreatic fibrosis by inducing TGF-β.[29] Moreover, previous studies have pinpointed that TNF-α308G/A polymorphism, a genetic predisposition to influence TNF levels, is significantly associated with the risk of developing both psoriasis and CP.[34, 36] Accordingly, CP, like psoriasis, is also an immune-mediated disease, at least in part. Prior research has also shown that aberrant autoimmunity in patients with immunodeficiency disorders may predispose them to having gastrointestinal or pancreatic disorders and skin diseases, including psoriasis.[37–40] Thus, deficiencies in immunohomeostasis might be the link between psoriasis and chronic pancreatitis. Our data showed that the incidence and HR of CP were greater in patients with psoriasis than control subjects after adjusting for potential confounding factors. Moreover, the risk for CP increased in parallel with the severity of psoriasis (ATE propensity score-weighted model). The finding was in agreement with the aforementioned studies and suggested that the involvement of other factors intrinsically linked to psoriasis, such as dysregulation of the immune system, predisposed patients with psoriasis to the development of CP.

Further evaluation regarding the effect of antipsoriatic drugs on the new onset of CP in patients with psoriasis showed that exposure to NSAIDs and methotrexate were associated with a reduced HR than non-exposure. This result was consistent with previous studies. [41–42] Prior research had suggested that administration of NSAIDs may significantly reduce the risk of acute pancreatitis after retrograde endoscopic cholangiopancreatography, which was proposed to be caused by activation of chemokines after endoscopic maneuvers.[41–42] A recent study also showed that methotrexate may reduce inflammation-related cytokine levels in acute pancreatitis[43] and relieve disease progression. Moreover, methotrexate had been used as an alternative treatment in azathioprine and 6-mercaptopurine induced pancreatitis in patients with inflammatory bowel disease.[43] Cyclosporine, azathioprine, cyclophosphamide, and hydroxyurea have been reported to induce and exacerbate acute pancreatitis.[44–48] Consistent with these studies, our study found that psoriasis patients taking cyclosporine, azathioprine, cyclophosphamide, and hydroxyurea had an increased risk of CP, though the associations were not statistically significant, probably due to small number of patients who exposed to these drugs.

Acitretin therapy may cause changes in the serum lipid profile resulting in hyperlipidemia in some patients.[49–50] In clinical trials, with doses ranging from 10 to 75 mg/day of acitretin, elevation of triglyceride levels (defined as >20 mg/dL) were noted in 66% of patients.[50] However, only one case treated with acitretin of fatal fulminant pancreatitis has been reported.[50] In agreement with previous reports, our results showed that acitretin use was not associated with significantly increased risk of CP. A possible explanation is increases of serum triglycerides to levels associated with pancreatitis are not common during acitretin therapy.

We also investigated the risk conferred by psoriasis in different patient subgroups. The HRs for CP were significantly increased in all subgroups, including those with mild and severe psoriasis and those with and without PsA. However, the risks for CP in patients with PsA were not significantly higher than those without PsA. The results may be attributed to multiple factors. The more frequent use of NSAIDs for pain associated with arthritis in patients with PsA might at least partially reduce the risk of CP compared with that for patients without PsA. This was probably due to the fact that when the risk was already elevated, mainly owing to psoriasis, the presence of concomitant arthritis did not have any additional effect. It is also possible that patients with PsA might have higher frequencies of comorbidities in these subgroups, which attenuated the additional effects conferred by arthritis on the risks for CP.

Several important limitations of our study should be considered. First, the diagnoses of CP and psoriasis used in our study relied on administrative claims data. Patients may be underdiagnosed or overdiagnosed with CP, resulting in misclassification bias. However, nondifferential misclassification bias would bias the results toward the null. Moreover, as mentioned above, an internal validation system exists for the accuracy of each claim included in the NHIRD. Previous reports have also confirmed the reliability of the diagnosis accuracy of psoriasis and pancreatitis based on ICD-9 codes.[6, 18, 51] A recent study by Shen et al. also validated the diagnostic code of acute pancreatitis, showing a positive predictive value of 90.0% (95% CI, 79.2–96.2%).[52] To further validate the results, we used a more rigorously defined outcome (e.g., CP with compatible orders of preceding imaging or laboratory examinations) in the sensitivity analysis and found results similar to those of the primary analyses (**Table B in S1 File**). Second, the NHRID does not provide some covariables such as behavior risk factors (the amount of daily alcohol consumption and tobacco use) due to the inherent limitation of the database. However, some of these unmeasured confounders may have been controlled by including alcohol dependence, tobacco use disorder, chronic obstructive pulmonary disease, and other comorbidities as an alternative covariable in the analyses. Third, we relied on treatment with systemic therapies or phototherapy as a surrogate marker for severe disease. However, it is possible that physicians are less likely to give systemic medications to patients with severe psoriasis with coexisting medical illnesses out of concern for systemic adverse effects. It is likely that there is misclassification with regard to psoriasis severity as with all epidemiologic studies. However, the reliability and validity of using these methods for grouping severe psoriasis has been demonstrated in previous studies.[8, 53–56] Lastly, more frequent hospital visits by patients with psoriasis than the general population may result in potential surveillance bias as they would have easier access to examinations, which might overestimate the risk of CP related to psoriasis. However, laboratory examinations (amylase/lipase) or imaging modalities for CP, were not a routine procedure performed during the therapeutic monitoring of psoriasis.

In conclusion, our nationwide study demonstrated that patients with psoriasis are at a significantly elevated risk of CP and the risk increased with severity of psoriasis. CP was thought to have a complex aetiology and the increased development of CP in patients with psoriasis was multifaceted and may be the result of several ongoing processes, including chronic inflammation in psoriasis, drugs, autoimmune pancreatitis, and genetic and behavioral risk factors. These results suggest the need for physicians to be aware of the pancreatic comorbidity associated with psoriasis and that earlier detection and intervention may reduce the significant morbidity and mortality in CP. Future studies are needed to better explore the pathophysiological basis underlying the relationship between psoriasis and CP, and to investigate the efficacy of systemic anti-inflammatory therapy in decreasing the risk of CP in patients with psoriasis.

## Supporting Information

S1 File **Table A**. Hazard ratios for chronic pancreatitis in patients with and without (controls) psoriasis derived from different Cox proportional hazard models. **Table B.** Sensitivity analyses.
(DOCX)
