# Supplementary material for: The Risk of Chronic Pancreatitis in Patients with Psoriasis: A Population-Based Cohort Study
Source: PLoS One. 2016 Jul 28;11(7):e0160041. doi: 10.1371/journal.pone.0160041 (PMC4965214; doi:10.1371/journal.pone.0160041)
Supplement: S1 File — Table A. Hazard ratios for chronic pancreatitis in patients with and without (controls) psoriasis derived from different Cox proportional hazard models. Table B. Sensitivity analyses. (DOCX) [file pone.0160041.s001.docx]

**Table A.** **Hazard ratios for chronic pancreatitis**. Hazard ratios for chronic pancreatitis in patients with and without (controls) psoriasis derived from different Cox proportional hazard Models

|  | **All psoriasis** | | **Arthritis** | | **Non-Arthritis** | | **Mild Psoriasis** | | **Severe psoriasis** | |
| --- | --- | --- | --- | --- | --- | --- | --- | --- | --- | --- |
|  | Stable_ATE | ATT | Stable_ATE | ATT | Stable_ATE | ATT | Stable_ATE | ATT | Stable_ATE | ATT |
| **Model 1** |  |  |  |  |  |  |  |  |  |  |
| Adjusted | 1.67 (1.41-1.98)^‡^ | 1.63 (1.30-2.05)^‡^ | 1.06 (0.72-1.56) | 1.04 (0.65-1.66) | 1.90 (1.57-2.30)^‡^ | 1.86 (1.43-2.42)^‡^ | 1.79 (1.48-2.17)^‡^ | 1.75 (1.36-2.26)^‡^ | 1.25 (0.83-1.88) | 1.20 (0.72-2.01) |
| **Model 2** |  |  |  |  |  |  |  |  |  |  |
| Adjusted | 1.89 (1.61-2.22)^‡^ | 1.31 (1.05-1.62)^‡^ | 1.44 (1.03-2.00)^†^ | 0.76 (0.48-1.19) | 2.06 (1.72-2.48)^‡^ | 1.57 (1.23-2.02)^‡^ | 2.06 (1.71-2.47)^‡^ | 1.74 (1.35-2.24)^‡^ | 2.37 (1.66-3.37)^‡^ | 5.17 (2.29-11.66)^‡^ |

Abbreviations: aHR, adjusted hazard ratio; ATE: average treatment effect; HR: hazard ratio; CI: confidence interval

†p < 0.05 for comparison between patients with and without psoriasis.

^‡^p < 0.001 for comparison between patients with and without psoriasis..

Model 1 is adjusted for gender, age group, and all comorbidities listed.

Model 2 is adjusted for gender, age group, medications, and all comorbidities listed.

| **Table B.** **Sensitivity analyses**. Defining the outcome of chronic pancreatitis by stringent criteria. | | | | | | |
| --- | --- | --- | --- | --- | --- | --- |
|  | **CPs** | **Follow-up (PY)** | | **Incidence  (1000 PY)** | **HR  (95% CI)** | **aHR^a^  (95% CI)** |
|  | ***n* (%)** | **Total** | **Mean** |  |  |  |
| **Primary model** | | | | | | |
| Psoriasis patients (n=48,430) | 196(0.40%) | 318,729 | 6.58 | 0.61 | 1.81 (1.53-2.15)^‡^ | 1.76 (1.47-2.10)^‡^ |
| Controls (n=193,720) | 433 (0.22%) | 1,276,332 | 6.59 | 0.34 | 1 (reference) | 1 (reference) |
| **Primary model with modified definition of the outcome^b^** | | | | | | |
| Psoriasis patients (n=48,430) | 173 (0.36%) | 318,813 | 6.58 | 0.54 | 2.08 (1.73-2.50)^‡^ | 2.03 (1.67-2.45)^‡^ |
| Controls (n=193,720) | 333 (0.17%) | 1,276,643 | 6.59 | 0.26 | 1 (reference) | 1 (reference) |
| ^a^ Model 2 is adjusted for gender, age group, medications, and all comorbidities listed.  ^b^ Refers to having ICD-9-CM code of chronic pancreatitis plus at least one of the following: undergoing amylase/lipase exams within 6 months prior to the diagnosis of chronic pancreatitis or receiving medical imaging studies such as abdominal ultrasonography, computed tomography, or magnetic resonance imaging within 6 months before and after the diagnosis of chronic pancreatitis.  †p < 0.05^‡^p < 0.001 for comparison between patients with psoriasis and without psoriasis. | | | | | | |
